# Tumour necrosis factor-α levels are elevated in adolescent patients with juvenile idiopathic arthritis on etanercept therapy

**DOI:** 10.1186/1546-0096-12-S1-P128

**Published:** 2014-09-17

**Authors:** Anna Radziszewska, Corinne Fisher, Linda Suffield, Geevithan Kumaran, Debajit Sen, Yiannis Ioannou

**Affiliations:** 1 Arthritis Research UK Centre for Adolescent Rheumatology at University College London, Great Ormond Street Hospital and UCLH, University College London, London, United Kingdom

## Introduction

The use of etanercept, a tumor necrosis factor (TNF) inhibitor, has revolutionized the treatment of juvenile idiopathic arthritis (JIA). TNF is a key cytokine implicated in the pathogenesis of inflammatory arthritis and etanercept, which is a soluble TNF receptor fusion protein, binds and inactivates TNF-α and lymphotoxin-A.

## Objectives

The aim of this study was to profile serum levels of TNF-α in a large cohort of adolescent patients with JIA.

## Methods

Serum TNF-α was measured in samples derived from 200 adolescent and young adult patients with JIA attending the adolescent and young adult rheumatology clinic at University College London Hospital using a commercial enzyme linked immunosorbent assay (ELISA) kit (eBioscience). Samples were tested in duplicate. Median age at sampling and median disease duration were 18 years and 8 years 9 months, respectively. Male:female ratio was 1:1.2. Equal numbers of patients with polyarticular (n=64) and enthesitis related arthritis (ERA, n=64) were tested in addition to 48 with oligoarticular, 16 systemic onset, and 8 psoriatic arthritis. Erythrocyte sedimentation rate (ESR) and C-reactive protein (CRP) measurements were also collected. Furthermore, an L929 cell viability bioassay was used to determine if the addition of etanercept abrogates the cytotoxic effects of TNF-α in L929 cells.

## Results

Surprisingly, TNF-α serum levels were shown to be markedly elevated in patients on etanercept (median TNF-α on etanercept= 134.2pg/ml, IQR [49.4-207.1], median not on etanercept = 4.2pg/ml, IQR [1.4-11.0], p<0.0001). TNF-α levels were also higher in patients on etanercept compared to those on other biologics (adalimumab, infliximab, abatacept, or tocilizumab, median= 4.4pg/ml, IQR [1.8-9.1]) or disease modifying anti-rheumatic drugs alone (median = 4.2 pg/ml, IQR [1.1-12.9]), p<0.0001. In addition, ESR and CRP levels had a negative correlation with high TNF-α levels in patients on etanercept (p=0.0018 and p=0.0034 respectively). Etanercept was included at its therapeutic serum concentration (2.4ug/ml) to ensure there was no cross reactivity with the assay. Finally, we showed that the addition to TNF-α to human serum leads to cytotoxicity in a TNF-α sensitive cell line, while adding etanercept at its therapeutic concentration along with TNF-α significantly reduces cell death (p = 0.0277).

## Conclusion

Patients treated with etanercept have higher levels of TNF-α. As the majority of patients with elevated TNF-α on etanercept were in remission, it is likely that this circulating TNF is biologically inactive. This is supported by our *in vitro* experiments in which the cytotoxic effect of TNF-α was abrogated upon addition of etanercept. Our hypothesis is that etanercept prolongs the half-life of circulating TNF-α. Further studies are needed to confirm these findings and dissect the mechanisms involved. As the association between high TNF-α and etanercept treatment is so strong, we hypothesise that it may be possible to measure TNF-α levels as a surrogate marker of adherence to this drug in this cohort of patients where adherence to medication can be a significant problem. This is a hypothesis that warrants further investigation.

## Disclosure of interest

None declared.

